# MicroRNA-206 expression levels correlate with clinical behaviour of rhabdomyosarcomas

**DOI:** 10.1038/sj.bjc.6605684

**Published:** 2010-05-25

**Authors:** E Missiaglia, C J Shepherd, S Patel, K Thway, G Pierron, K Pritchard-Jones, M Renard, R Sciot, P Rao, O Oberlin, O Delattre, J Shipley

**Affiliations:** 1 Molecular Cytogenetics Team, The Institute of Cancer Research, Sutton, Surrey SM2 5NG, UK; 2 Department of Histopathology, Royal Marsden NHS Trust, London SW3 6JJ, UK; 3 Institut Curie, Paris 75248, France; 4 Section of Paediatrics, The Institute of Cancer Research, Sutton, Surrey SM2 5NG, UK; 5 Department of Paediatrics, University Hospital, Catholic University of Leuven, Leuven 3000, Belgium; 6 Department of Pathology, University Hospital, Catholic University of Leuven, Leuven 3000, Belgium; 7 Whitehead Institute for Biomedical Research, Cambridge, MA 02142, USA; 8 Department of Paediatric Oncology, Institut Gustave-Roussy, Villejuif Cedex 94805, France; 9 INSERM, U830, Génétique et Biologie des Cancers, Paris 75248, France

**Keywords:** rhabdomyosarcoma, microRNA, overall survival, expression profile, cell line

## Abstract

**Background:**

Rhabdomyosarcomas (RMSs) are primarily paediatric sarcomas that resemble developing skeletal muscle. Our aim was to determine the effects of microRNAs (miRNA) that have been implicated in muscle development on the clinical behaviour of RMSs.

**Methods:**

Expression levels of miR-1, miR-206, miR-133a and miR-133b were quantified by RT–PCR in 163 primary paediatric RMSs, plus control tissues, and correlated with clinico-pathological features. Correlations with parallel gene expression profiling data for 84 samples were used to identify pathways associated with miR-206. Synthetic miR-206 was transfected into RMS cell lines and phenotypic responses assessed.

**Results:**

Muscle-specific miRNAs levels were lower in RMSs compared with skeletal muscle but generally higher than in other normal tissues. Low miR-206 expression correlated with poor overall survival and was an independent predictor of shorter survival in metastatic embryonal and alveolar cases without *PAX3/7-FOXO1* fusion genes. Low miR-206 expression also significantly correlated with high SIOP stage and the presence of metastases at diagnosis. High miR-206 expression strongly correlated with genes linked to muscle differentiation and low expression was associated with genes linked to MAPkinase and NFKappaB pathway activation. Increasing miR-206 expression in cell lines inhibited cell growth and migration and induced apoptosis that was associated with myogenic differentiation in some, but not all, cell lines.

**Conclusion:**

miR-206 contributes to the clinical behaviour of RMSs and the pleiotropic effects of miR-206 supports therapeutic potential.

Rhabdomyosarcoma (RMS) are a heterogenous group of sarcomas that account for over half the cases of soft tissue sarcomas in children (Slater and Shipley, 2007). Although the cure rate for patients with localised disease is around 70%, the presence of metastasis is associated with a much poorer prognosis (Breneman *et al*, 2003; Oberlin *et al*, 2008). In addition, current treatment modalities confer significant morbidity and less toxic treatments are urgently needed (Stevens, 2005). Better stratification for current protocols is also required, potentially incorporating the molecular characteristics of RMS (Oberlin *et al*, 2008; Davicioni *et al*, 2009). Histologically, there are two major subtypes: embryonal (ERMS), which account for 60–70% of cases, and alveolar (ARMS). Approximately 70% of ARMS harbour fusion genes resulting from 5′ sequences of *PAX3* or *PAX7* genes fusing to 3′ sequences of *FOXO1* (Galili *et al*, 1993; Shapiro *et al*, 1993; Davis *et al*, 1994). Alveolar cases with *PAX3-FOXO1* are characterised by metastatic behaviour and a poor prognosis. No such molecular marker is predictive of clinical behaviour in the embryonal subtype. Although the cell of origin is currently unknown, (Linardic *et al*, 2005; Mercado and Barr, 2007) the defining characteristic of RMS is that they show myogenic features and are composed of cells that fail to undergo terminal differentiation (Merlino and Helman, 1999).

MicroRNAs (miRNAs) are small (20–22 nt) RNA molecules that function by negatively regulating the stability or translational efficiency of their target mRNAs, most commonly by base-pairing to partially complementary sequences within the 3′ UTR (Kim, 2005; Esquela-Kerscher and Slack, 2006). miRNAs are involved in diverse biological processes including apoptosis, cellular proliferation and differentiation. It is noteworthy that they have shown oncogenic or tumour suppressor roles in different cancers types and miRNA profiles can identify diagnostic/prognostic characteristics (Calin *et al*, 2002; Lu *et al*, 2005; Roldo *et al*, 2006; Volinia *et al*, 2006; Yanaihara *et al*, 2006). It is suggested that miRNAs represent novel targets for therapy (Mishra and Merlino, 2009; Petri *et al*, 2009).

A role for miRNAs in the myogenic programme has been identified. miR-1 and miR-206 are essential for the formation of differentiated skeletal muscle cells (Sokol and Ambros, 2005; Zhao *et al*, 2005; Chen *et al*, 2006; Kim *et al*, 2006; Rao *et al*, 2006). Although miR-206 is almost exclusively expressed in skeletal muscle, miR-1 is also highly expressed in heart muscle and, to a lesser extent, in other tissues including bladder and prostate (Baskerville and Bartel, 2005; Kim *et al*, 2006; Liang *et al*, 2007). miR-133a and miR-133b have been associated with myoblast proliferation (Chen *et al*, 2006). In addition, miR-1 and miR-206 have been shown to be expressed at a lower level in RMS compared with normal skeletal muscle (Subramanian *et al*, 2008; Wang *et al*, 2008; Taulli *et al*, 2009; Yan *et al*, 2009). However, these studies were performed on small populations of patient samples not suitable to identify correlations between miRNA expression levels and clinico-pathological features. Recently, increasing expression of miR-1 and miR-206 in two RMS cell lines was shown to induce myogenic differentiation and, for miR-206, inhibition of cell line growth (Taulli *et al*, 2009; Yan *et al*, 2009). Significantly, the degree of myogenic differentiation in RMS has been inversely related to proliferation and migration (Lollini *et al*, 1991; Merlino and Helman, 1999; Barlow *et al*, 2006; Davicioni *et al*, 2009).

We have analysed a large series of primary RMS samples for miR-1, miR-206, miR-133a and miR-133b expression levels to identify correlations with clinical behaviour. In view of recent results emphasising the biological and clinical effect of fusion gene status in RMS, including our own survival analyses (Davicioni *et al*, 2006, 2009; Williamson *et al*, 2010), we have performed comparisons considering either all samples or samples divided by their fusion status (either positive or negative for *PAX3/7-FOXO1*). Strong correlations were identified with miR-206 levels that clinically stratify embryonal and alveolar fusion gene-negative RMS. Comparison with parallel expression profiling data and functional analyses after modulating miR-206 levels in cell lines identified likely biological mechanisms underlying the clinical behaviour. Furthermore, the various tumour suppressive effects of increasing miR-206 described here and elsewhere are consistent with therapeutic opportunities for RMS patients.

## Materials and methods

### Primary tumour samples

In total, 163 primary RMS tumours samples from resection or biopsy material and 15 normal skeletal muscle tissues were snap frozen and collected from different centres. Total RNA was extracted as previously described (Williamson *et al*, 2005). RNA from normal lung, colon, brain and thyroid was commercially available (Clontech, Mountain View, CA, USA). All primary tumours were pathologically reviewed and their fusion gene status determined (Williamson *et al*, 2005). This study had ethical approval (local research ethics committee protocol no. 1836, multi-regional research ethics committee/98/4/023) and, wherein required, consent had been obtained. All patients were treated similarly with multi-agent chemotherapy and surgery, with or without radiotherapy for local control. High-dose therapy with bone marrow or stem-cell rescue in first remission was limited to high-risk patients. Patients were registered to, or treated according to, the MMT 89 (Stevens, 2005), MMT 95 (Defachelles *et al*, 2009) and EpSSG RMS 05 protocols for localised RMS and MMT IV 89/91 or MMT 98 (Bergeron *et al*, 2008; Oberlin *et al*, 2008) for metastatic RMS. The clinico-pathological information and correlations with overall survival, including previously defined parameters, (Oberlin *et al*, 2008) are summarised in Table 1 and Supplementary Table 1.

### Cell lines

Human cell lines derived from ERMS and ARMS were used in this study. The sources of these, their culture conditions and DNA fingerprint data used for identity verification are summarised in Supplementary Table 2. A primary culture of human myoblasts was also available.

### Quantitative real-time PCR for miRNA detection

The TaqMan miRNA assay was used according to the manufacturer's instructions to measure the expression of miR-1, miR-133a, miR-133b and miR-206 using pre-developed reagents from ABI (Applied Biosystems, Carlsbad, CA, USA) run on an ABI 7900HT Real-Time PCR machine. U6 small nuclear RNA (RNU6B) and small nucleolar RNA, C/D box 48 (RNU48) were used as endogenous controls to normalise the data. Analysis was performed by the comparative threshold cycle (Ct) method accordingly to User Bulletin no. 2 (Applied Biosystems). Results were expressed as ΔCt in comparison with the average expression of the two endogenous controls and all experiments were performed in triplicate. As the distribution of miRNA expression was not found to be normal (Shapiro–Wilk normality test), differential expression between subgroups was performed using non-parametric tests namely Mann–Whitney *U-*test and Kruskal–Wallis rank sum test. Log-rank test and Cox's proportional hazards regression were used to test the correlation of categorical or continuous parameters to overall or disease-free survival. For this latter analysis, we considered the time to the first progression of the disease (either as local relapse or distant metastasis). Patients who died without going through clinical stabilisation of their disease were censored at their death. The expression of miRNAs showed a heavy tailed distribution (particularly the left) with only 3–4 Cts difference within samples between the 25th and 75th percentiles. Therefore, miRNA expression levels were categorised using quartiles with expression within the first quartile defined as ‘low’, ‘med’ when expression was in the second and third quartiles and otherwise ‘high’. Multivariate survival analysis was performed using Cox's proportional hazard model with ‘med’ and ‘high’ collapsed into one category. The covariates included in the analysis were evaluated using both forward and reverse stepwise methods.

### Correlations with gene expression profiles

Expression profiling was generated using Affymetrix HGU133Plus2 chips (Santa Clara, CA, USA) following manufacturer's instructions. Eighty-four samples from patients with TaqMan miR-206 measurements (Table 1) were analysed after normalisation using *gcrma* package that included further samples described elsewhere (Williamson *et al*, 2010). Probesets were filtered based on their expression, removing those showing a level <6 (as log2 intensity) in all samples. Linear regression analysis and Pearson's product moment correlation coefficients were calculated dividing patient samples on the basis of their fusion gene status. Probesets that showed a correlation with miR-206 expression levels >0.55 and <–0.5 with a *P*-value <0.001 were selected for data mining analysis. Gene ontology was performed using *GOstats* package using the hypergeometric test to identify association of biological process terms. All analysis was performed using R-2.9 software. Ingenuity Pathway Analysis Software (Ingenuity, Redwood City, CA, USA) was also used to identify relevant networks and pathways over-represented in our gene list. A score was computed for each network that reflects the negative logarithm of the *P-*value and indicates the likelihood of genes in a network being found together.

### miRNA transfections and cell proliferation assay

Cells were plated at 4 × 10^3^–6 × 10^3^ cells per well in 96-well plates in sextuplicates and, after 24 h, transfected using HiPerfect reagent with 25 nM of synthetic miR-206 (Qiagen GmbH, Hilden, Germany or Ambion Inc., Austin, TX, USA) or a negative control (AllStars Negative (Qiagen GmbH) or pre-miR negative (Ambion Inc.). Transfection efficiency was tested using a FITC labelled oligo. Cell proliferation of RD and RH30 cells were assessed over a 4-day time-course and measured using the CyQuant NF kit (Invitrogen, Carlsbad, CA, USA) according to manufacturer's instructions. RUCH3 and RH41 cell viability was assessed at 96 h after transfection using the CellTitre 96 Aqeuous One solution (Promega, Madison, WI, USA) according to the manufacturer's instructions. All measurements from the sextuplicates were averaged.

### Cell cycle and apoptosis analyses

In all, 1 × 10^5^cells were plated in a six-well plate and transfected as described above. Cells were harvested at 72 h after transfection, stained with propidium iodide and analysed by FACS using a standard protocol (http://www.flemingtonlab.com/Protocols/PreparingCellsforFACS-PI.pdf). Cell cycle distribution was evaluated using FlowJo software and Dean/Jett/Fox algorithm (Tree Star Inc., Ashland, OR, USA). Apoptosis was evaluated in cells grown in 96-well plates by measuring caspase-3 and -7 activation 72 h after transfection using Caspase-Glo 3/7 Assay (Promega) according to the manufacturer's protocol. All experiments were performed in triplicate.

### *In vitro* migration assays

Cells were plated in six-well plates and transfected as described above. At 72 or 96 h after transfection, 2.5 × 10^4^ cells in DMEM 1% FCS were placed in triplicate into cell culture inserts (BD, Franklin Lakes, NJ, USA) and submerged into specially adapted 24-well plates (BD) containing 500 *μ*l DMEM 10% FCS. Non-migrated cells were removed 24 h later and the base of the inserts fixed in 100% methanol and stained with 2% crystal violet solution. Cells were photographed at × 10 magnification (four fields of view) and counted manually. All experiments were performed in triplicate.

### Western blot and antibodies

Cells were washed once with ice-cold PBS and lysed *in situ* with Cell Lysis Buffer (Cell Signaling, Danvers, MA, USA) according to the manufacturer's instructions. Protein was quantified using the BCA Protein Assay Kit (Thermo Scientific, Waltham, MA, USA) according to the manufacturer's instructions. Protein (8–10 *μ*g) was resolved on 4–12% 1.5 mm Bis-Tris gels (Invitrogen) and transferred to PDVF membranes (Invitrogen). Protein was visualised using the ECL Plus western blotting detection system (GE Healthcare, Little Chalfont, Bucks, UK) and a ChemiDoc XRS chemiluminescent detection system (Bio-Rad Inc., Hercules, CA, USA). Anti-MYOG (F5D) and -MET (code 18321) antibodies were from the Developmental Studies Hybridoma Bank, maintained by the University of Iowa (Iowa City, IA, USA), and IBL (Gunma, Japan), respectively.

## Results

### Muscle-specific miRNAs are underexpressed in RMS compared with normal skeletal muscle

**Figure 1:**
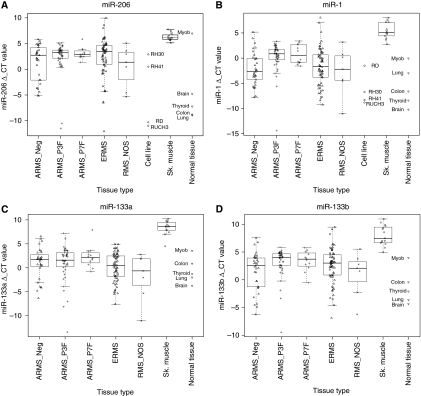
Box and whiskers plots representing the expression of (**A**) miR-206, (**B**) miR-1, (**C**) miR133a and (**D**) miR133b in 33 ARMS fusion negative (ARMS_Neg), 45 ARMS *PAX3-FOXO1A* (ARMS_P3F), 12 ARMS *PAX7-FOXO1A* (ARMS_P7F), 66 ERMS, 7 RMS not otherwise specified (RMS_NOS), 4 RMS cell lines, 15 normal skeletal muscles (Sk.muscle), 1 myoblasts sample (Myob) and 4 normal tissues. Δ_CT values were calculated by subtracting miRNA CT values from the average CT values of two endogenous controls (RNU6B and RNU48).

The expression of muscle-specific miRNAs was quantified in 163 primary RMS samples, 4 RMS cell lines, one myoblast sample, 15 normal skeletal muscle and 4 other normal tissues (lung, colon, brain and thyroid) by quantitative RT–PCR. All miRNAs showed a statistically significant lower level of expression in RMS compared with the skeletal muscle samples (Wilcox test, *P*<0.001) (Figure 1). With the exception of miR133a, a higher level of expression was observed in tumours compared with other normal tissue. In addition, fusion gene negative ERMS and ARMS showed a similar wide range of expression for all the miRNAs measured. miR-1 was significantly higher in the ARMS fusion positive compared with all other RMS (Figure 1 and Supplementary Table 3).

### miR-206 expression correlates with overall survival in RMS

**Figure 2:**
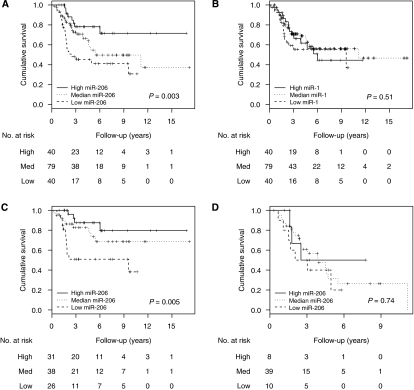
Kaplan–Meier plots for overall survival with (**A**) miR-206 expression (**B**) miR-1 expression in all RMS samples and (**C**) miR-206 within fusion gene-negative patients and (**D**) miR-206 within fusion gene-positive patients. Expression levels within the first quartile was considered ‘low’, ‘med’ when expression was between the second and third quartile and ‘high’ when within the top quartile. *P*-values were obtained using log-rank test.

Only the expression of miR-206 was significantly inversely correlated with overall survival when tested as a continuous value using a Cox proportional hazard model (*N*=159, HR 0.93 (95% Cl 0.87–0.98), *P*=0.018). The Kaplan–Meier curves show the correlation between overall survival and expression of miR-206 when categorised into three groups using expression level whereas this is not the case for miR-1 or miR-133a/b (Table 2, Figure 2B and Supplementary Table 4). It is noteworthy that lower miR-206 expression was significantly associated with shorter survival in ERMS and ARMS fusion gene-negative patients (Figures 2A and C, Table 2). This was not found in patients with ARMS positive for *PAX3/7-FOXO1* (Figure 2D and Supplementary table 4). No correlation was observed between muscle-specific miRNAs expression and disease-free survival (data not shown). However, a significantly higher number of patients relapsed without leading to death when miR-206 expression was high or median compared with patients with low expression (high, 22% (9 out of 40); medium, 18% (14 out of 79); and low, 10% (4 out of 40); *χ*^2^ test *P*<0.001). In addition, more patients with low miR-206 expression died of progressive disease, which was not stabilised clinically and in which there was no disease-free survival to be measured, compared with those with median or high expression (low, 12% (5 out of 40); medium, 4% (3 out of 79); and high, 2% (1 out of 40); *χ*^2^ test *P*<0.001).

In multivariate analysis miR-206 expression was retained in the best model (Table 3) when using all RMS samples, although this did not reach the conventional threshold for statistical significance. Similarly, a multivariate analysis was also performed for fusion gene-negative patients. However, the presence of only 26 events within this group strongly limited the number of clinico-pathological parameters evaluated in association with miR-206 expression. Nevertheless, expression within the first quartile retained its independent predictive value for shorter overall survival in fusion gene-negative cases when combined with either the presence of bone–bone-marrow or any other metastasis at diagnosis (Supplementary Table 5). Furthermore, miR-206 expression was significantly lower in patients with higher SIOP stage and metastasis at diagnosis (Supplementary Table 3 and Supplementary Figures 1A and B). When divided by their fusion gene status, only fusion-negative patients still showed a significant correlation with these parameters (Supplementary Figures 1C–F).

### Expression of miR-206 in primary tumours correlates with gene expression associated with multiple pathways, including myogenic differentiation

Gene expression profiling data were analysed for 84 samples that were also tested for miR-206 expression. Within the fusion gene-negative samples 149 and 117 known genes showed a significant positive or negative correlation with miR-206 expression levels, respectively. Only 3 and 44 genes passed the inclusion criteria in the fusion gene-positive group as the range of expression in this group was narrow (Supplementary Table 6).

Data mining of the lists of genes associated with high miR-206 expression in fusion-negative patients performed using Gene Ontology and Ingenuity Software clearly showed strong correlates to genes associated with muscle differentiation (Supplementary Table 7 and supplementary Figure 2). Low expression of miR-206 in fusion-negative RMS was associated with genes involved MAPkinase and NF-KappaB signalling as well as IL-4 that is involved in the immune response.

### Transfection of miR-206 in RMS cell lines shows anti-tumourigenic effects

**Figure 3:**
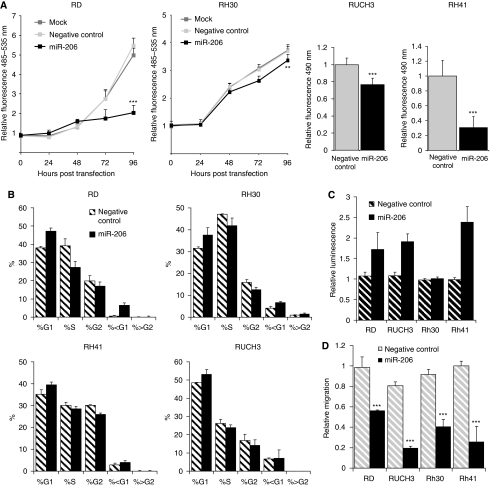
Overexpression of miR-206 in RMS cells reduces cell proliferation, cell cycle progression and migration and enhances apoptosis: (**A**) proliferation and cell viability of RMS cells, (**B**) cell cycle delay in G_0_G_1_, (**C**) apoptosis in all but RH30 cells and (**D**) reduction in cell migration ^***^P<0.001 and ^**^P<0.01.

To further analyse the role of miR-206, ERMS and ARMS cell lines were transfected with a miR-206 mimic or a negative control. Transfection efficacy was always above 80%, and miR-206 levels were comparable to those observed in skeletal muscle (data not shown). Cell growth and viability were significantly reduced in all cell lines with a cell cycle delay in G1/G0 phase (Figures 3A and B). Apoptosis was detected through activation of caspase3/7 (Figure 3C) and confirmed as an increase in the sub G1/G0 cell population. Cell lines also showed reduction in their ability to migrate after transfection of miR-206 (Figure 3D). Elevated miR-206 was linked with a more elongated and differentiated cell phenotype associated with increased levels of myogenin mRNA and protein in cell lines RD, RH30 and RH41 but not RUCH3 (Supplementary Figures 3A and B and data not shown). Expression of the tyrosine kinase receptor MET is associated with migratory behaviour and has been shown to be a target of miR-206 (Christensen *et al*, 2005; Yan *et al*, 2009). Decreased expression of MET protein was observed at 72 and 96 h after transfection (Supplementary Figure 3C).

## Discussion

In this study, we investigated the clinical relevance of expression levels of the muscle-specific miRNAs miR-1, miR-206, miR-133a and miR-133b in a large population of primary RMS samples. This revealed an inverse correlation between miR-206 levels and adverse outcome in fusion gene-negative cases that is consistent with underlying molecular mechanisms associated with miR-206 expression levels.

The expression of muscle-specific miRNAs was markedly reduced in RMS compared with normal terminally differentiated skeletal muscle. A broad range of expression levels was observed but these were generally higher than in other normal tissues tested. In light of evidence for their role in promoting skeletal muscle differentiation (Kim *et al*, 2006), it is likely that the low but detectable levels of miR-1 and miR-206 expression contribute to the arrested myogenic phenotype of this tumour. Previous analyses of small series of RMS have also shown low levels of miR-1 and miR-206 relative to skeletal muscle (Subramanian *et al*, 2008; Wang *et al*, 2008; Taulli *et al*, 2009; Yan *et al*, 2009) but undetectable levels by quantitative RT–PCR are described (Taulli *et al*, 2009), which was not the case in our study. miR-1 was significantly more highly expressed in the fusion gene-positive ARMS compared with the ERMS or fusion gene-negative ARMS. This is consistent with the biological and clinical similarities of ERMS and fusion gene-negative ARMS (Davicioni *et al*, 2006, 2009; Williamson *et al*, 2010). Higher miR-1 levels in fusion gene-positive cases is also consistent with the fact that ARMS usually show higher expression of muscle-specific markers such as myogenin than ERMS (Morotti *et al*, 2006).

Survival analyses revealed significant correlations between miR-206 expression levels and overall survival within fusion gene-negative patients. The lack of correlation with disease-free survival was attributable to low expression of miR-206 not increasing the probability of relapse, but an association with more aggressive tumours. In fact, we also show that low miR-206 expression significantly correlated with high SIOP stage and the presence of metastases at diagnosis. It is noteworthy that in the fusion gene-negative cases, miR-206 expression was an independent predictor for shorter overall survival when associated with presence of metastasis or bone/marrow metastasis at diagnosis. miR-206 levels in fusion gene-negative RMS may have clinical implications because ERMS currently lack molecular markers indicative of poor outcome and such patients could benefit from treatment intensification.

Several lines of evidence suggest that these clinical correlations are linked to the differentiation status of RMS and the propensity of undifferentiated cells to migrate and potentially metastasise. Comparing the gene expression profiling data for primary tumours with their parallel miR-206 expression levels revealed that miR-206 expression strongly correlated with expression of markers of muscle differentiation, including myogenin. This is consistent with a shift in the gene expression profile of RD18 towards differentiated muscle after miR-206 induction recently observed by (Taulli *et al*, 2009). Similarly, we also observed induced expression of myogenin after transfection of miR-206 mimics in three of four cell lines. Interestingly, low levels of miR-206 correlated with genes associated with networks centred on NF-KappaB, ERK and JNK signalling pathways, which are considered to have a role in RMS development (Wang *et al*, 2007, 2008; Durbin *et al*, 2009; Marampon *et al*, 2009). Overexpression of genes associated with immune response and inflammation were also highlighted. IL-4 signalling was one of the top canonical pathways identified and activation of the IL-4 pathway has recently been shown to inhibit myogenin expression and increase the migratory ability of RMS cells (Nanni *et al*, 2009).

All four cell lines modulated to overexpress miR-206 showed a reduction of proliferation and migration, associated with reduced MET expression, a known target of miR-206 (Taulli *et al*, 2009; Yan *et al*, 2009). However, one cell line did not show any associated signs of muscle differentiation. miR-206 has been shown to act as a tumour suppressor in the Hela cell line without a link to induction of muscle differentiation (Song *et al*, 2009). miR-206 has also been associated with metastatic and oestrogen-positive breast cancers (Adams *et al*, 2007; Kondo *et al*, 2008; Tavazoie *et al*, 2008). Therefore, miR-206 likely exerts roles distinct from inducing skeletal muscle differentiation in RMS potentially in a cell context-specific manner. These multiple roles could explain why miR-206, and not the other muscle-related miRNAs tested, showed a statistical correlation with survival data. Analysis of genes inversely correlated with low miR-1 expression (data not shown) showed, unlike those for miR-206, strong associations with a signature for high proliferation. Similarly, miR133a/b has been specifically associated with myoblast proliferation (Chen *et al*, 2006). Proliferation rate *per se* may not relate to adverse patient outcome with current therapies.

*PAX3/7-FOXO1-*positive tumours showed no correlations between miR-206 levels and outcome data. They had a smaller range of expression levels for miR-206 than fusion gene-negative RMS and therefore appeared a more homogenous miR-206-expressing group. PAX3-FOXO1 is a potent transcription factor that affects on the tumourigenic and myogenic phenotype of RMS (Mercado and Barr, 2007; Kikuchi *et al*, 2008). In spite of the lack of clinical correlations with miR-206 expression levels within this fusion gene-positive group, introduction of miR-206 into cell lines expressing *PAX3-FOXO1* has been shown here and elsewhere (Taulli *et al*, 2009) to have therapeutic potential. Together with our results showing the expression and clinical correlations of miR-206 in primary RMS, this may lead to novel therapeutic strategies.

## Figures and Tables

**Table 1:** Clinical-pathological data of the patients involved in this study and their association with overall survival

**Parameter**	**No. of patients in TaqMan analysis**	**No. of patients in microarray analysis**
*Histology*		
ERMS	66 (41%)	31 (37%)
ARMS	90 (55%)	53 (63%)
PAX3-FOXO1 fusion gene-positive	45	30
PAX7-FOXO1 fusion gene-positive	12	8
Fusion gene-negative	33	15
RMS not otherwise specified	7 (4%)	0
		
Total	163	84
		
Gender (M/F)	91/72	47/37
		
Median age	5 (0–23)	5.5 (0–21)
		
*Primary tumour location*		
Favourable	58	32
Unfavourable	79	50
Unknown	26	2
		
*SIOP stage*		
Stage I	46	25
Stage II	44	26
Stage III	21	9
Stage IV	43	22
Unknown	9	2
		
*Metastasis*		
Present at diagnosis	43	22
Absent at diagnosis	108	61
Unknown	12	1
		
*Median survival*		
Median follow-up time	4.0 y^a^	
		
Overall survival	9.6 y	
*N* of events (*N* patients)	61 (159)	
		
Event-free survival	2.8 y	
*N* of events (*N* patient)	79 (159)	

Abbreviations: ARMS=alveolar RMS; ERMS=embryonal RMS; RMS= rhabdomyosarcoma.

a On the basis of patients with censored data.

**Table 2:** Correlation of miR-1, 133a, 133b and 206 with overall survival

	**All RMS patients**			**Fusion-negative patients**		
**miRNA**	**No. of patients**	**HR (95% CI**)	**Log-rank test (*P*)**	**No. of patients**	**HR (95% CI)**	**Log-rank test (*P*)**
*miR-206 expression*						
High miR-206	40	1	0.003	31	1	0.005
Med miR-206	79	2.1 (0.9–4.7)		38	2.0 (0.6–6.5)	
Low miR-206	40	3.7 (1.7–8.4)^**^		26	4.9 (1.6–15.0)^**^	
						
*miR-1 expression*						
High miR-1	40	1	0.515	16	1	0.229
Med miR-1	79	0.9 (0.5–1.6)		45	0.9 (0.5–1.6)	
Low miR-1	40	1.2 (0.6–2.5)		34	1.2 (0.6–2.5)	
						
*miR-133a expression*						
High miR-133a	40	1	0.859	21	1	0.314
Med miR-133a	79	1.2 (0.6–2.2)		40	0.9 (0.3–2.9)	
Low miR-133a	40	1.2 (0.6–2.4)		34	1.7 (0.6–4.9)	
						
*miR-133b expression*						
High miR-133b	40	1	0.355	21	1	0.161
Med miR-133b	79	1.2 (0.6–2.3)		44	1.4 (0.4–4.3)	
Low miR-133b	40	1.6 (0.8–3.3)		30	2.5 (0.8–7.8)	

Abbreviations: CI=confidence interval; HR=Hazard Ratio; miRNA=microRNA; RMS= rhabdomyosarcoma.

^**^P<0.01.

**Table 3:** Multivariate analysis using Cox proportional hazard model in all RMS sample

**Parameter^a^**	**No. of samples^b^**	**HR (CI 95%) OS**	**OS *P*-value**
*Primary tumour location*			0.002
Favourable	50	1	
Unfavourable	69	3.1 (1.45–7.33)	
			
*Bone or bone marrow metastasis*			0.002
No	101	1	
Yes	18	3.3 (1.65–6.78)	
			
*Fusion gene*			0.009
None	74	1	
PAX7-FOXO1	10	1.2 (0.37–3.71)	
PAX3-FOXO1	35	3.0 (1.43–6.17)	
			
*miR-206 expression*			0.078
High	96	1	
Low	23	1.9 (0.96–3.87)	

Abbreviations: ARMS=alveolar RMS; CI=confidence interval; ERMS=embryonal RMS; OS=overall survival; RMS= rhabdomyosarcoma.

a Parameters used in the stepwise Cox proportional hazard model: primary tumour location (favourable, unfavourable), presence of metastasis at diagnosis, presence of bone or bone marrow metastasis at diagnosis, age (favourable <10 years, unfavourable), fusion gene (none, PAX7-FOXO1, PAX3-FOXO1), histology (ERMS, ARMS), miR-206 expression (low, high). In particular, miR-206 expression was defined low if within the first quartile and high otherwise.

b The stepwise analysis was performed on 116 patients who had information for all the parameters considered in our model as well as the follow-up, whereas the final coefficient values were calculated using the number of patients reported in each column.

## Supplementary Material

Supplementary Figure 1

Supplementary Figure 2a,b
