# Supplementary figures and images for: MicroRNA-206 expression levels correlate with clinical behaviour of rhabdomyosarcomas
Source: Br J Cancer. 2010 May 25;102(12):1769–77. doi: 10.1038/sj.bjc.6605684 (PMC2883695; doi:10.1038/sj.bjc.6605684)

Supplementary figure 1

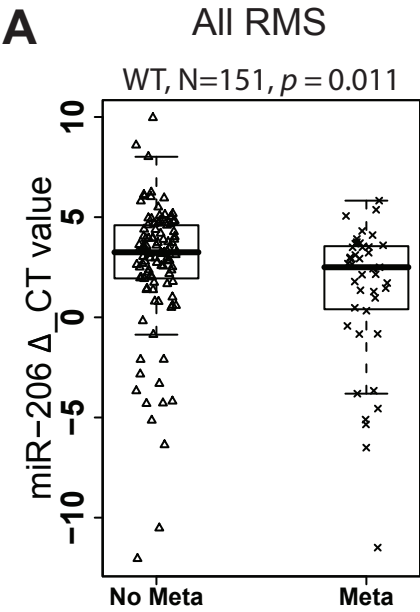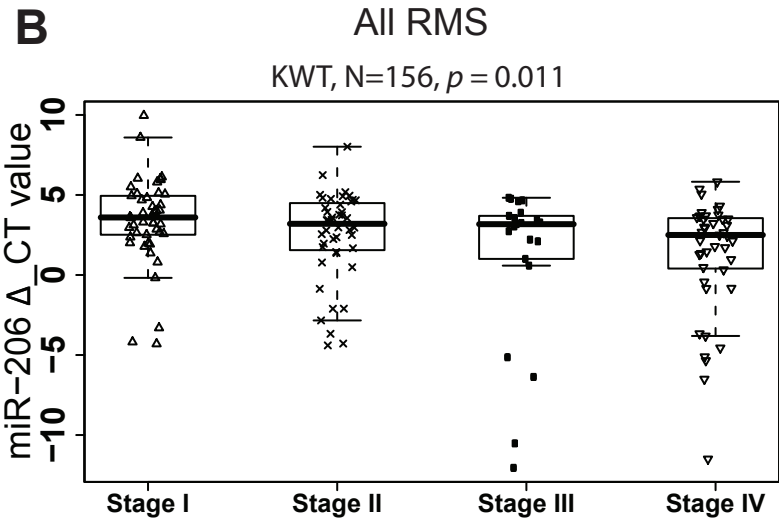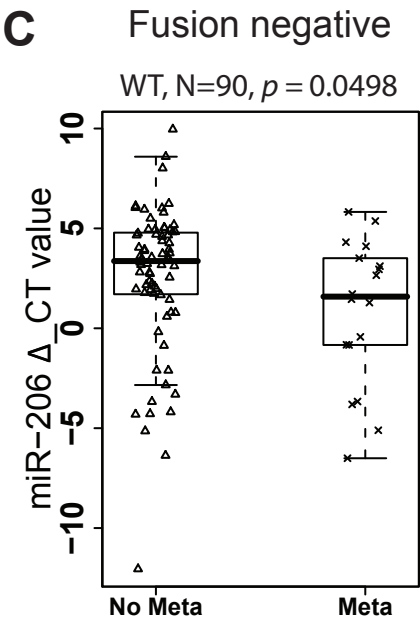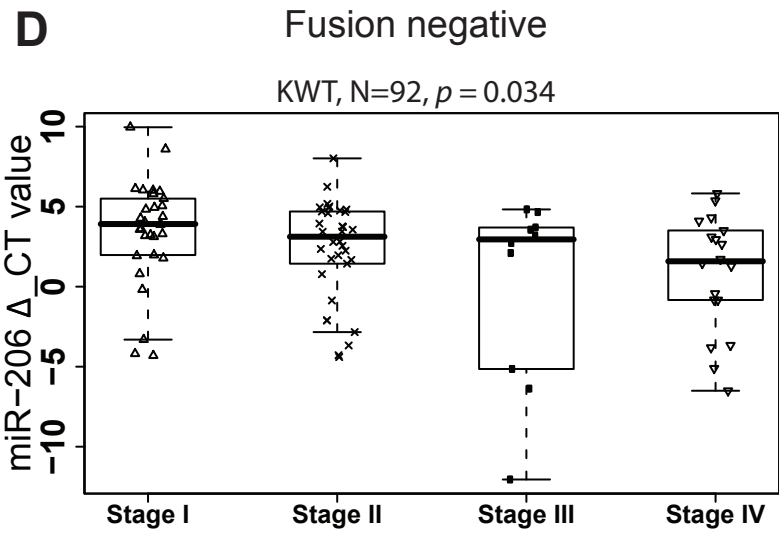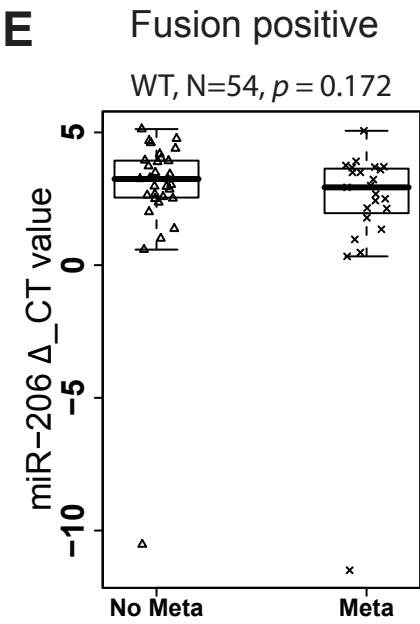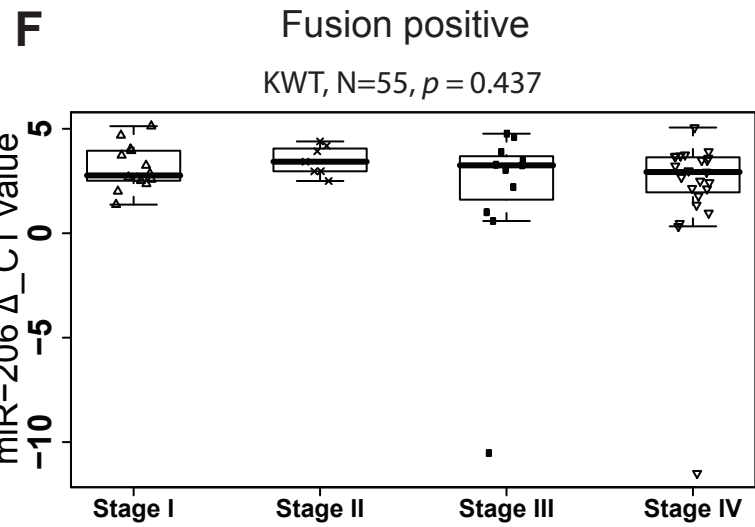

Supplement: Supplementary Figure 1 [file 6605684x1.pdf]

Supplementary figure 2

A

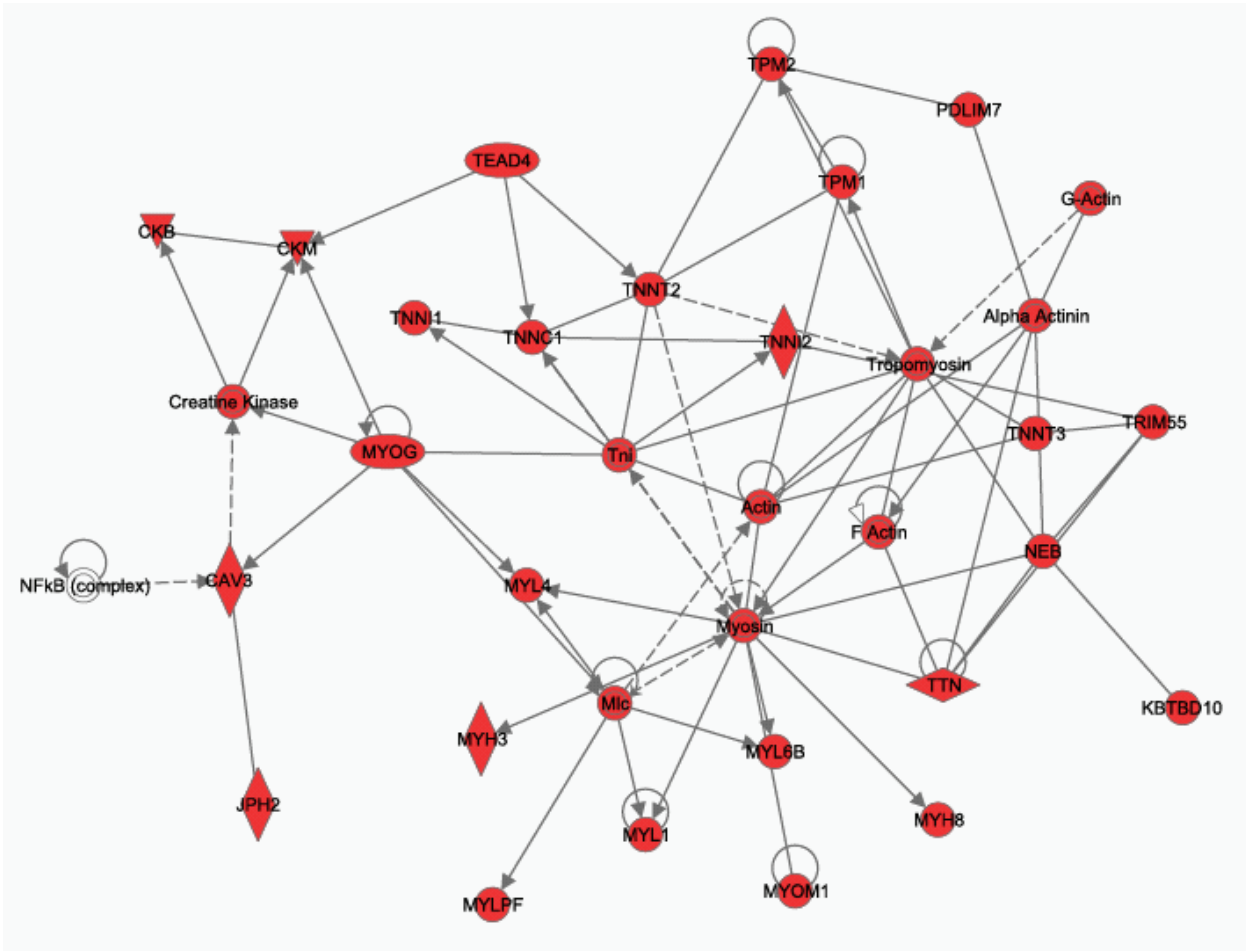

B

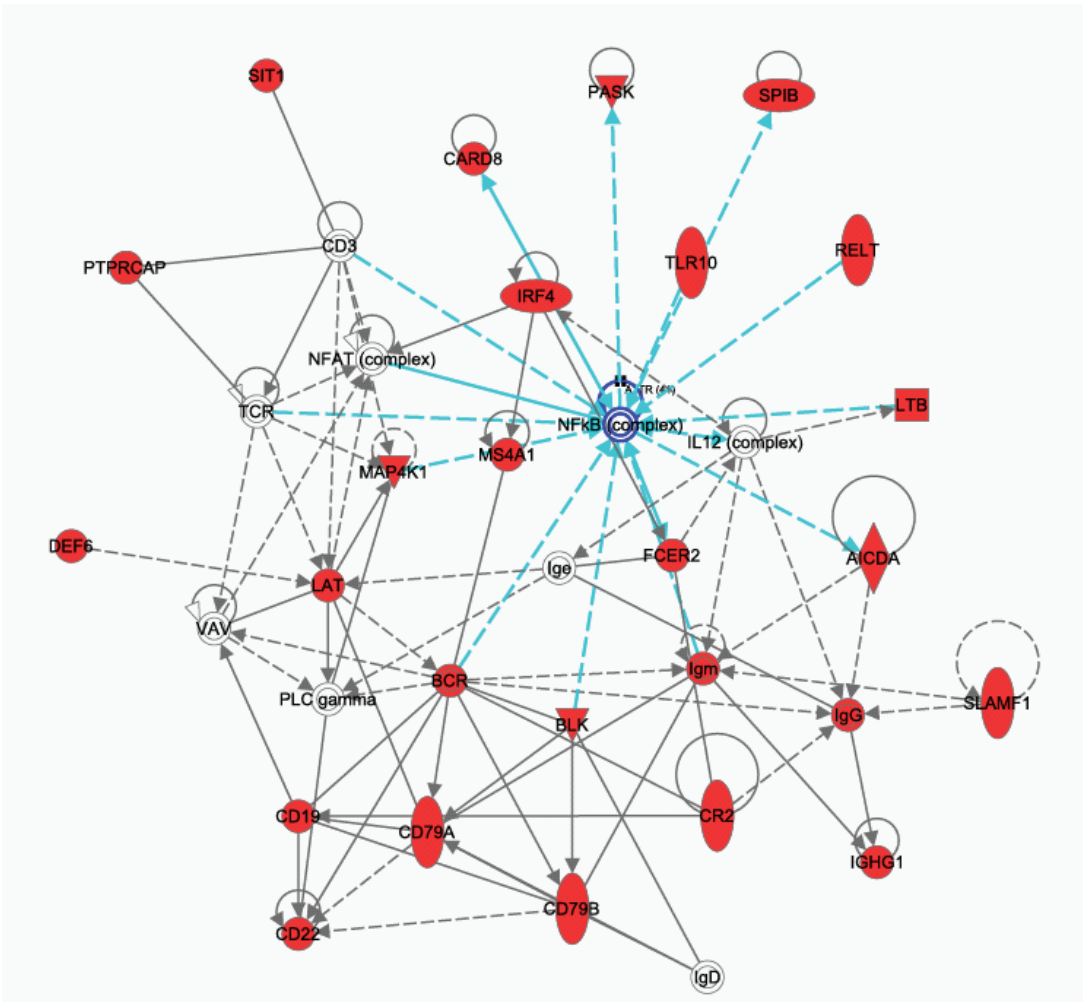

Supplement: Supplementary Figure 2a,b [file 6605684x2.pdf]

**C**

**C**

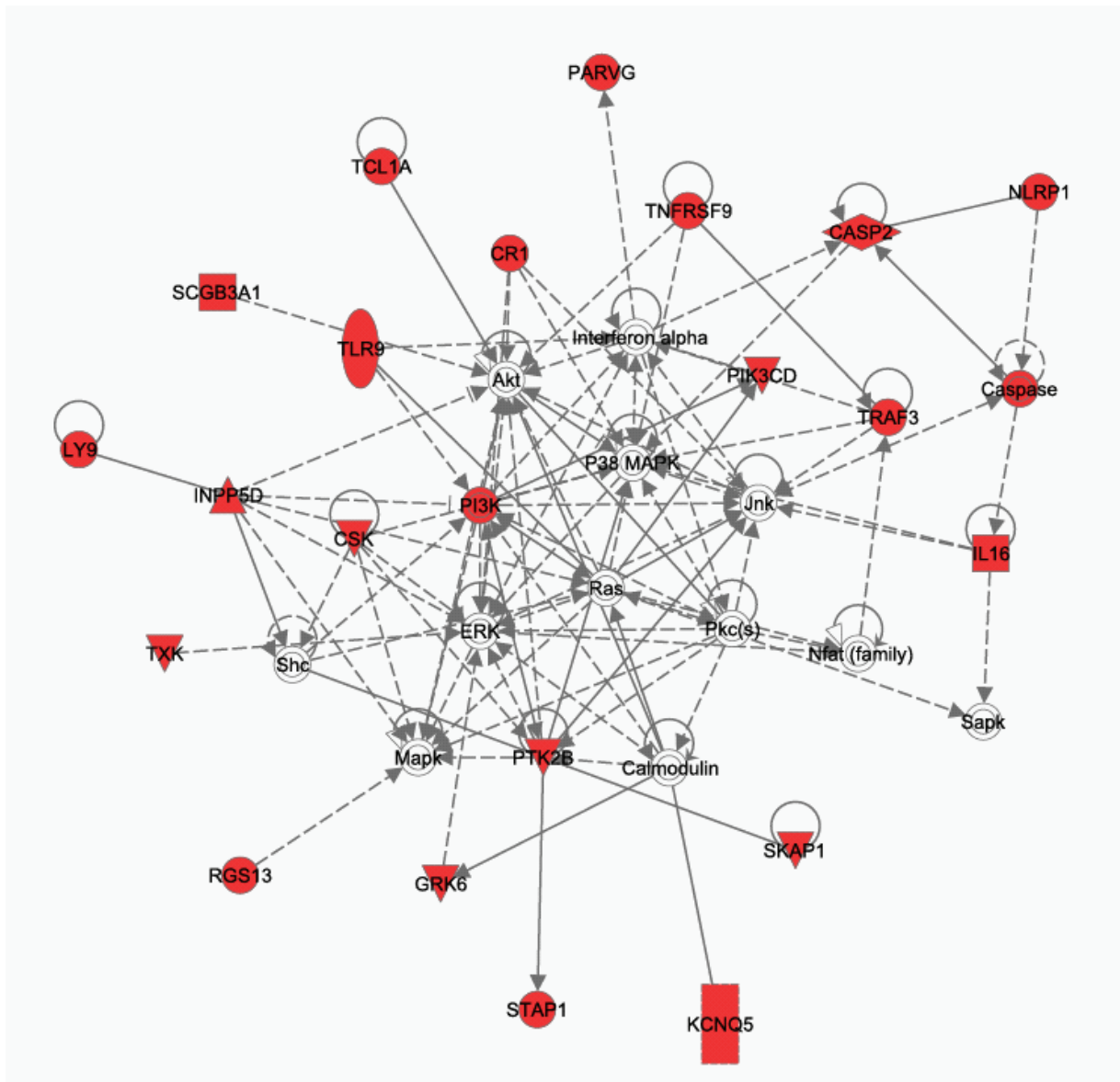

Supplement: Supplementary Figure 2c [file 6605684x3.pdf]

Supplementary figure 3

A

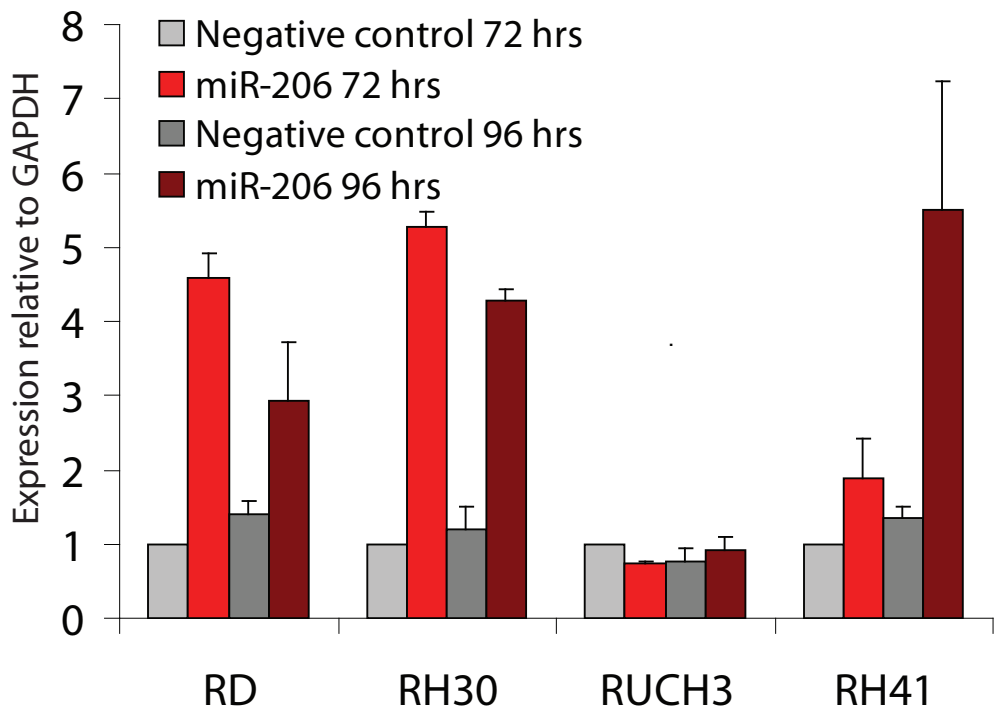

B

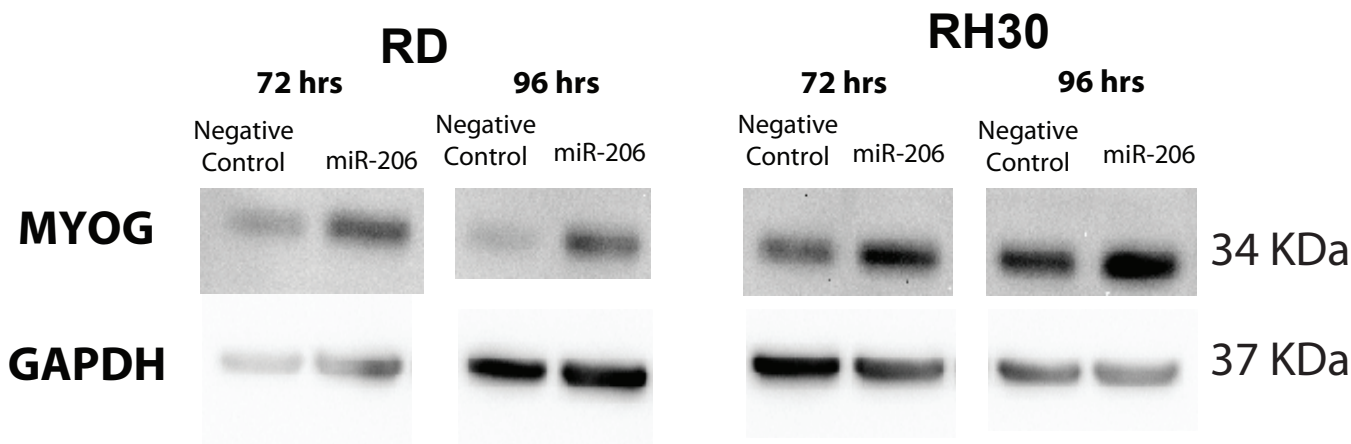

C

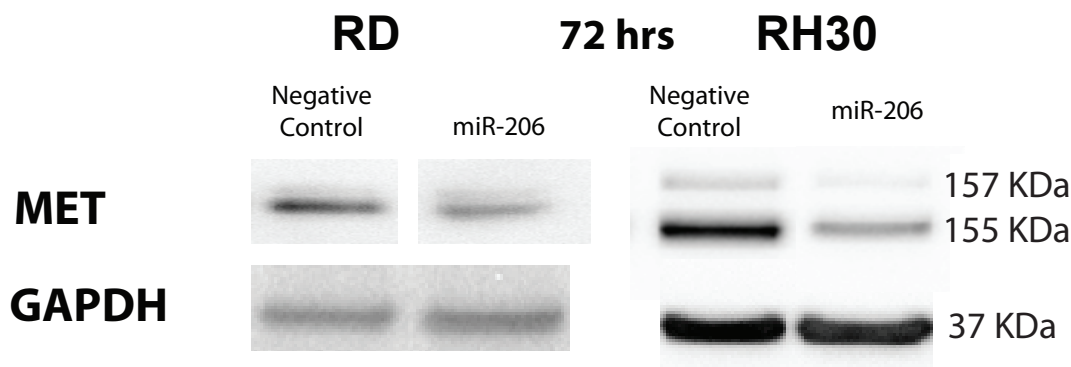

Supplement: Supplementary Figure 3 [file 6605684x4.pdf]
