# Supplementary material for: MicroRNA-206 expression levels correlate with clinical behaviour of rhabdomyosarcomas
Source: Br J Cancer. 2010 May 25;102(12):1769–77. doi: 10.1038/sj.bjc.6605684 (PMC2883695; doi:10.1038/sj.bjc.6605684)
Supplement: Supplementary Figure Legends [file 6605684x5.doc]

**Supplementary figure legends**

**Supplementary figure 1.** Box and whiskers plots representing the expression levels of miR-206 according to metastatic status and SIOP stage in all RMS samples (**A,B**), in fusion negative patients (**C,D**) and in fusion positive patients (**E,F**). The expression of miR-206 is significantly different between metastatic and not metastatic and within SIOP stages considering all the RMS samples or fusion negative patients. No such difference is observed in fusion positive samples. MiR-206 expression was measured by quantitative RT-PCR and expressed as Δ_CT values relative to an average value of two endogenous controls (RNU6B and RNU48). **WT**: Wilcoxon rank sum test; **KWT**: Kruskal-Wallis rank sum test; **N**: number of patients.

**Supplementary figure 2.** Representative Ingenuity pathway analyses of genes correlating with high and low levels of miR-206 expression in RMS samples: Significant networks involved in skeletal muscle differentiation were identified in high miR-206 expressing RMS and immune and inflammatory response pathways in low miR-206 expressing RMS. (**A**) A significant network associated to genes correlating positively with miR-206 patients. Markers of myogenic differentiation (myogenin and myosin) are central to the molecular relationships between molecules. (**B,C**) Two networks generated with genes correlating inversely with miR-206 expression in patients (colour in red). These networks had functions associated with immune response, hematological system development and inflammation. NF-KappaB is central to genetic interactions in **B** and ERK, JNK, P38 MAPK are central to interactions in **C**. The lines connecting the molecules define the molecular relationships, with dashed and solid lines indicating an indirect or direct (physical contact between molecules) interaction, respectively.

**Supplementary figure 3.** MiR-206 re-expression induces MYOG protein and RNA expression in RMS cells and down-regulates MET. Protein and RNA was extracted from RMS cells transfected with 25nM miR-206 mimic at 72 and 96 hours post transfection. MYOG is increased at the mRNA (**A**) and protein level (**B**) in RMS cells. No changes in MYOG expression were identified in RUCH3. MYOG expression was measured by TaqMan assay using Standard curve method and GAPDH as the endogenous control. (**C**) MET protein is decreased in RD and RH30 cells at 72 hours post transfection with miR-206.
