# Supplementary material for: MicroRNA-206 expression levels correlate with clinical behaviour of rhabdomyosarcomas
Source: Br J Cancer. 2010 May 25;102(12):1769–77. doi: 10.1038/sj.bjc.6605684 (PMC2883695; doi:10.1038/sj.bjc.6605684)
Supplement: Supplementary Table 4 [file 6605684x9.doc]

**Supplementary Table 4:** Correlation of miR-1, 133a, 133b and 206 with overall survival in fusion positive patients

|  | **Fusion positive patients** | | |
| --- | --- | --- | --- |
| **miRNA** | **N of patients** | **HR (95% CI)** | **Log Rank test (P)** |
| ***miR-206 Expression*** |  |  |  |
|  |  |  |  |
| High miR-206 | 8 | 1 | 0.742 |
| Med miR-206 | 39 | 1.2 (0.4 -4.1) |  |
| Low miR-206 | 10 | 1.6 (0.4 -6.3) |  |
|  |  |  |  |
| ***miR-1 Expression*** |  |  |  |
|  |  |  |  |
| High miR-1 | 23 | 1 | 0.086 |
| Med miR-1 | 31 | 0.9 (0.4 -2.0) |  |
| Low miR-1 | 3 | 3.5 (1.0 -12.9) |  |
|  |  |  |  |
| ***miR-133a Expression*** |  |  |  |
|  |  |  |  |
| High miR-133a | 17 | 1 | 0.404 |
| Med miR-133a | 38 | 1.0 (0.4 -2.3) |  |
| Low miR-133a | 2 | 2.6 (0.6 -12.7) |  |
|  |  |  |  |
| ***miR-133b Expression*** |  |  |  |
|  |  |  |  |
| High miR-133b | 18 | 1 | 0.985 |
| Med miR-133b | 31 | 1.0 (0.4 -2.3) |  |
| Low miR-133b | 8 | 1.1 (0.4 -3.3) |  |
|  |  |  |  |
