# Supplementary material for: MicroRNA-206 expression levels correlate with clinical behaviour of rhabdomyosarcomas
Source: Br J Cancer. 2010 May 25;102(12):1769–77. doi: 10.1038/sj.bjc.6605684 (PMC2883695; doi:10.1038/sj.bjc.6605684)
Supplement: Supplementary Table 5 [file 6605684x10.doc]

**Supplementary Table 5:** Multivariate analysis using Cox Proportional Hazard Model in fusion negative RMS sample.

**A**

| **Parameter** | **N of**  **samples** | **HR (CI 95%) OS** | **OS p value** |
| --- | --- | --- | --- |
|  |  |  |  |
| **Metastasis at diagnosis** |  |  | 0.008 |
| No | 72 | 1 |  |
| Yes | 18 | 3.2 (1.36-7.78) |  |
|  |  |  |  |
| **miR-206 expression** |  |  | 0.025 |
| High | 66 | 1 |  |
| Low | 24 | 2.5 (1.12-5.61) |  |
|  |  |  |  |

**B**

| **Bone or bone marrow metastasis** |  |  | 0.001 |
| --- | --- | --- | --- |
| No | 76 | 1 |  |
| Yes | 6 | 6.3 (2.02-19.41) |  |
|  |  |  |  |
| **miR-206 expression** |  |  | 0.01 |
| High | 63 | 1 |  |
| Low | 19 | 3.2 (1.33-7.92) |  |
|  |  |  |  |

Because of the low number of events within fusion negative patients, it was not possible to evaluate a full model with all the parameters. However, the independency of miR-206 expression in predicting overall survival in this subset of patients was tested in association with the presence of metastasis (**A**) and bone or bone marrow metastasis at diagnosis (**B**) using two separate models. These two parameters showed the highest association with overall survival at univariate level within fusion negative patients (supplementary table 1). MiR-206 expression was categorized as low if within the 1^st^ quartile evaluated in all RMS samples.
